# High-Acuity Alcohol-Related Complications During the COVID-19 Pandemic

**DOI:** 10.1001/jamahealthforum.2024.0501

**Published:** 2024-04-12

**Authors:** Bryant Shuey, Alyssa Halbisen, Matthew Lakoma, Fang Zhang, Stephanie Argetsinger, Emily C. Williams, Benjamin G. Druss, Hefei Wen, J. Franklin Wharam

**Affiliations:** 1 Department of Population Medicine, Harvard Pilgrim Health Care Institute, Harvard Medical School, Boston, Massachusetts; 2 Now with Center for Research on Health Care, Division of General Internal Medicine, Department of Medicine, University of Pittsburgh, Pittsburgh, Pennsylvania; 3 Department of Health Systems and Population Health, University of Washington School of Public Health, Seattle; 4 Health Services Research and Development, Center of Innovation for Veteran-Centered and Value-Driven Care, Veterans Affairs Puget Sound Health Care System, Seattle, Washington; 5 Rollins School of Public Health, Emory University, Atlanta, Georgia; 6 Department of Medicine, Duke University, Durham, North Carolina; 7 Duke-Margolis Institute for Health Policy, Durham, North Carolina

## Abstract

**Importance:**

Research has demonstrated an association between the COVID-19 pandemic and increased alcohol-related liver disease hospitalizations and deaths. However, trends in alcohol-related complications more broadly are unclear, especially among subgroups disproportionately affected by alcohol use.

**Objective:**

To assess trends in people with high-acuity alcohol-related complications admitted to the emergency department, observation unit, or hospital during the COVID-19 pandemic, focusing on demographic differences.

**Design, Setting, and Participants:**

This longitudinal interrupted time series cohort study analyzed US national insurance claims data using Optum’s deidentified Clinformatics Data Mart database from March 2017 to September 2021, before and after the March 2020 COVID-19 pandemic onset. A rolling cohort of people 15 years and older who had at least 6 months of continuous commercial or Medicare Advantage coverage were included. Subgroups of interest included males and females stratified by age group. Data were analyzed from April 2023 to January 2024.

**Exposure:**

COVID-19 pandemic environment from March 2020 to September 2021.

**Main Outcomes and Measures:**

Differences between monthly rates vs predicted rates of high-acuity alcohol-related complication episodes, determined using claims-based algorithms and alcohol-specific diagnosis codes. The secondary outcome was the subset of complication episodes due to alcohol-related liver disease.

**Results:**

Rates of high-acuity alcohol-related complications were statistically higher than expected in 4 of 18 pandemic months after March 2020 (range of absolute and relative increases: 0.4-0.8 episodes per 100 000 people and 8.3%-19.4%, respectively). Women aged 40 to 64 years experienced statistically significant increases in 10 of 18 pandemic months (range of absolute and relative increases: 1.3-2.1 episodes per 100 000 people and 33.3%-56.0%, respectively). In this same population, rates of complication episodes due to alcohol-related liver disease increased above expected in 16 of 18 pandemic months (range of absolute and relative increases: 0.8-2.1 episodes per 100 000 people and 34.1%-94.7%, respectively).

**Conclusions and Relevance:**

In this cohort study of a national, commercially insured population, high-acuity alcohol-related complication episodes increased beyond what was expected in 4 of 18 COVID-19 pandemic months. Women aged 40 to 64 years experienced 33.3% to 56.0% increases in complication episodes in 10 of 18 pandemic months, a pattern associated with large and sustained increases in high-acuity alcohol-related liver disease complications. Findings underscore the need for increased attention to alcohol use disorder risk factors, alcohol use patterns, alcohol-related health effects, and alcohol regulations and policies, especially among women aged 40 to 64 years.

## Key Points

**Question:**

Was the COVID-19 pandemic associated with increased rates of high-acuity alcohol-related complications?

**Findings:**

In this cohort study of a US national, commercially insured population, high-acuity alcohol-related complication episodes increased beyond what was expected in 4 of 18 pandemic months. Women aged 40 to 64 years experienced increases of 33.3% to 56.0% in high-acuity complication episodes in 10 of 18 pandemic months, a pattern associated with a large and sustained increase in high-acuity alcohol-related liver disease complications.

**Meaning:**

Findings underscore the need for increased attention to alcohol use disorder risk factors, alcohol use patterns, alcohol-related health effects, and alcohol regulations and policies, especially among women aged 40 to 64 years.

## Teaser

This cohort study assesses trends in people with high-acuity alcohol-related complications admitted to the emergency department, observation unit, or hospital during the COVID-19 pandemic, focusing on demographic differences.

## Introduction

Alcohol use and alcohol-related deaths increased over the past decade, with mortality rising faster among women than men.^1,2^ Alcohol use complications are highest between the fourth and sixth decades of life, often due to alcohol-related liver disease (ALD) development.^3,4^ The COVID-19 pandemic was associated with increased alcohol consumption, particularly among women, likely due to social isolation and stress.^5^ While several studies have examined differences in alcohol-related hepatitis hospitalizations or alcohol-related mortality in 2020 compared with prior years,^2,5,6,7,8^ a rigorous analysis of the pandemic’s effect on a broader set of high-acuity alcohol-related complications is lacking. Such analyses could provide granular information about how socially disruptive phenomena such as the COVID-19 pandemic affect alcohol use–related presentations to the health system. We hypothesized that the pandemic would be associated with increased high-acuity alcohol-related complications caused by higher alcohol consumption and barriers to outpatient treatment services.

## Methods

### Study Design

We studied commercial claims data from Optum’s deidentified Clinformatics Data Mart database from March 2017 to September 2021. We conducted a longitudinal interrupted time series study of a rolling cohort aged 15 years and older with at least 6 months of continuous insurance enrollment. We measured presentations to the emergency department, observation unit, or hospital using claims-based algorithms and alcohol-specific *International Statistical Classification of Diseases and Related Health Problems, Tenth Revision,* diagnosis codes (eAppendix in Supplement 1). We defined high-acuity alcohol-related complication episodes as conditions representing acute decompensation of chronic alcohol-related diseases that necessitated urgent or emergent management by a medical professional (eAppendix and eTable 1 in Supplement 1).

The Duke University and Harvard Pilgrim Health Care Institute institutional review boards approved this study and waived need for informed consent because deidentified data were used. This report adheres to the Strengthening the Reporting of Observational Studies in Epidemiology (STROBE) reporting guidelines.

### Statistical Analysis

We ran regression models with generalized estimating equations, an identity link, and robust standard errors to compare observed monthly rates after March 2020 to rates predicted by the trajectory of the March 2017 to February 2020 baseline fitted trend (eAppendix in Supplement 1). Models adjusted for age group (15-39, 40-64, 65-74, and ≥75 years), sex, US division (9 divisions), poverty level of residence, and seasonality using quarterly indicators (eAppendix in Supplement 1). Additional analyses were stratified by age group and sex. We also separately analyzed rates of high-acuity ALD episodes over time. We describe COVID-19 pandemic era trends (April 2020 to September 2021) and present estimates of differences between monthly rates of high-acuity alcohol-related complication episodes vs predicted rates.

The statistical analysis was performed using Stata, version 16 (StataCorp), and SAS Studio software, release 3.7, enterprise edition (SAS Institute). Results are reported based on 2-sided tests of statistical significance, defined as *P* < .05.

## Results

**Figure 1.**
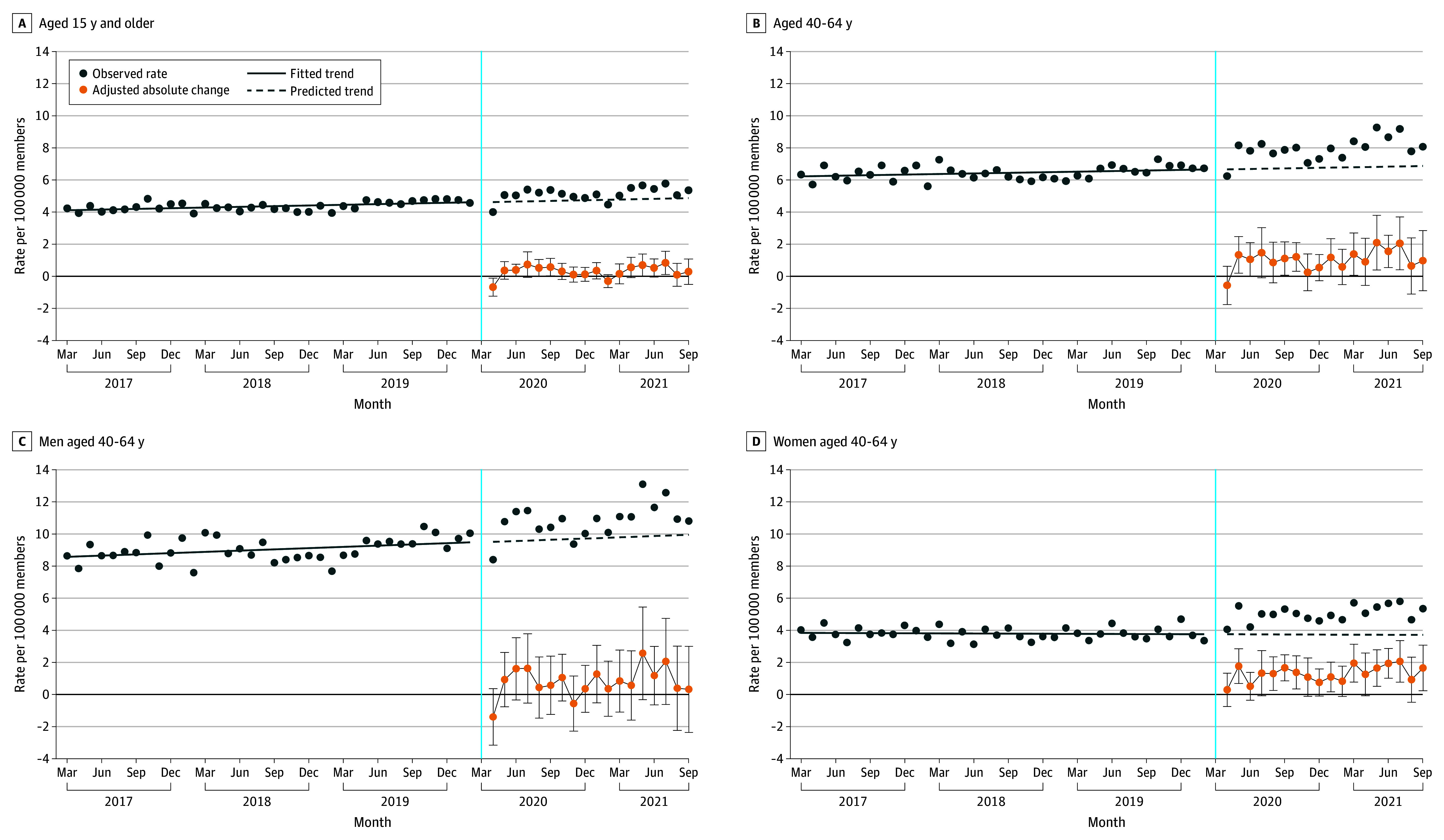
Monthly Rates of High-Acuity Alcohol-Related Complication Episodes Among a National Commercially Insured Population Fitted and predicted rates were generated using unadjusted segmented regression. Absolute change estimates and 95% CIs (error bars) were generated from regression model coefficients using nonlinear combinations of parameters adjusted for member-level age category, sex, US division, seasonality, and poverty level of residence based on 5-digit zip code. A, Complication episodes across all age groups comprise diagnoses representing acute decompensation of chronic alcohol-related diseases presenting to the emergency department, observation unit, or hospital such as acute alcohol-related hepatitis, alcohol-related cardiomyopathy, or alcohol gastritis with bleeding. Diagnoses were independently developed and cross-verified by 2 authors (B.S. and J.F.W.). There was 95% agreement with classifications, and differences were reviewed and reconciled. B, Adults aged 40 to 64 years are shown because this age category had high rates of alcohol-related morbidity and mortality. Age category was not included in the regression model for analyses depicted. C and D, Male and female adults aged 40 to 64 years are shown to highlight differences in high-acuity alcohol-related complications. Age category and sex were not included in the regression models for analyses depicted. The vertical blue line indicates the time of declaration of the COVID-19 public health emergency.

Study characteristics in April 2017, April 2020, and April 2021 are summarized in eTable 2 in Supplement 1. Among high-acuity diagnoses, 54% to 66% were ALD related, 29% to 39% were alcohol withdrawal or alcohol-related mood disorders, 3% to 5% were alcohol-related cardiomyopathy, and 1% to 3% were alcohol-related gastritis with bleeding (eTable 3 in Supplement 1). Other than a transient decrease in April 2020 and a reduction that was not statistically significant in February 2021, monthly rates of high-acuity alcohol-related complication episodes were higher than predicted in all follow-up months (Figure 1 and the Table), though only 4 of 18 reached statistical significance. The range of absolute and relative increases in these 4 months was 0.4 to 0.8 episodes per 100 000 people and 8.3% to 19.4%, respectively.

**Table.** Absolute and Relative Changes in Monthly Rates of High-Acuity Alcohol-Related Complication Episodes vs Predicted Rates During the April 2020 to September 2021 COVID-19 Pandemic Era^a^

Month	Change in composite high-acuity alcohol-related complications per 100 000 people							
	Aged ≥15 y		Aged 40-64 y		Men aged 40-64 y		Women aged 40-64 y	
	Absolute (95% CI)^b^	Relative %^c^	Absolute (95% CI)	Relative %	Absolute (95% CI)	Relative %	Absolute (95% CI)	Relative %
Apr 2020	−0.7 (−1.2 to −0.1)	−13.6	−0.6 (−1.8 to 0.6)	−6.3	−1.4 (−3.2 to 0.4)	−11.6	0.3 (−0.7 to 1.3)	8.1
May 2020	0.4 (−0.2 to 0.9)	9.0	1.3 (0.2 to 2.5)	22.2	0.9 (−0.8 to 2.6)	12.9	1.8 (0.7 to 2.8)	47.3
Jun 2020	0.4 (0 to 0.8)	8.3	1.1 (0 to 2.1)	16.9	1.6 (−0.3 to 3.5)	19.2	0.5 (−0.4 to 1.4)	12.2
Jul 2020	0.7 (−0.1 to 1.5)	15.7	1.5 (−0.1 to 3.0)	23.1	1.6 (−0.5 to 3.8)	19.5	1.3 (−0.1 to 2.7)	33.9
Aug 2020	0.5 (0 to 1.0)	11.3	0.9 (−0.4 to 2.1)	14.0	0.4 (−1.5 to 2.3)	7.2	1.3 (0.3 to 2.3)	33.3
Sep 2020	0.6 (0 to 1.1)	14.5	1.1 (0.1 to 2.1)	17.1	0.6 (−1.2 to 2.4)	8.0	1.7 (0.8 to 2.5)	42.3
Oct 2020	0.3 (−0.2 to 0.8)	9.0	1.2 (0.3 to 2.1)	18.9	1.1 (−0.4 to 2.5)	13.4	1.4 (0.3 to 2.4)	35.0
Nov 2020	0.1 (−0.4 to 0.6)	4.9	0.2 (−0.9 to 1.4)	4.7	−0.6 (−2.3 to 1.2)	−3.3	1.1 (−0.1 to 2.3)	27.1
Dec 2020	0.1 (−0.3 to 0.5)	2.9	0.5 (−0.3 to 1.4)	8.2	0.4 (−1.1 to 1.8)	3.2	0.7 (−0.1 to 1.6)	22.9
Jan 2021	0.3 (−0.2 to 0.9)	7.2	1.2 (0 to 2.3)	17.5	1.3 (−0.5 to 3.1)	12.6	1.1 (0.2 to 2.0)	32.1
Feb 2021	−0.3 (−0.7 to 0.1)	−6.3	0.6 (−0.5 to 1.7)	8.9	0.4 (−1.4 to 2.1)	3.3	0.8 (−0.1 to 1.8)	25.0
Mar 2021	0.2 (−0.5 to 0.8)	5.1	1.4 (0.1 to 2.7)	23.7	0.8 (−1.1 to 2.8)	13.1	2.0 (0.8 to 3.1)	53.3
Apr 2021	0.6 (−0.1 to 1.2)	14.7	0.9 (−0.6 to 2.4)	18.4	0.6 (−1.6 to 2.7)	12.7	1.3 (−0.1 to 2.6)	35.9
May 2021	0.7 (0 to 1.4)	17.7	2.1 (0.4 to 3.8)	35.9	2.6 (−0.3 to 5.5)	33.0	1.6 (0.5 to 2.8)	46.5
Jun 2021	0.5 (0 to 1.1)	12.8	1.6 (0.5 to 2.6)	26.7	1.2 (−0.6 to 3.0)	18.1	1.9 (1.0 to 2.9)	52.7
Jul 2021	0.8 (0.1 to 1.6)	19.4	2.1 (0.4 to 3.7)	34.0	2.1 (−0.6 to 4.7)	27.0	2.1 (0.8 to 3.4)	56.0
Aug 2021	0.1 (−0.6 to 0.8)	4.1	0.6 (−1.1 to 2.4)	13.5	0.4 (−2.2 to 3.0)	10.1	0.9 (−0.5 to 2.3)	25.4
Sep 2021	0.3 (−0.5 to 1.1)	10.1	1.0 (−0.9 to 2.9)	17.5	0.3 (−2.4 to 3.0)	8.6	1.6 (0.2 to 3.1)	44.0

^a^ Absolute and relative changes vs predicted rates are presented for people with high-acuity alcohol-related complications presenting to the emergency department, observation unit, or hospital. Models for absolute changes adjusted for member-level age category, sex, US division, seasonality, and poverty level of residence based on 5-digit zip code. Age category was not included in models stratified by age, and sex was and not included in models stratified by sex.

^b^ Absolute change estimates and 95% CIs were generated from adjusted regression model coefficients using nonlinear combinations of parameters.

^c^ Given that the adjusted relative changes are sensitive to the referent group chosen for each covariate in each model, unadjusted relative changes were calculated. Relative change estimates were calculated by dividing the unadjusted absolute change (unadjusted observed rate minus unadjusted predicted rate) by the unadjusted predicted rate for each pandemic month.

The subgroup of members aged 40 to 64 years had a similar but more pronounced pattern of pandemic-era increases. The magnitude of all monthly estimates after April 2020 was higher than predicted, with 9 reaching statistical significance. The range of absolute and relative increases in these 9 months was 1.1 to 2.1 episodes per 100 000 people and 16.9% to 35.9%, respectively. Women in this age group experienced statistically significant increases in 10 of 18 pandemic months, with a range of absolute and relative increases of 1.3 to 2.1 episodes per 100 000 people and 33.3% to 56.0%, respectively. Men in this age group did not experience statistically significant changes, though most pandemic-era estimates were positive in magnitude. Additional subgroup analyses by age group and sex revealed no consistent pandemic-era patterns and few statistically significant changes (eTable 4 in Supplement 1).

**Figure 2.**
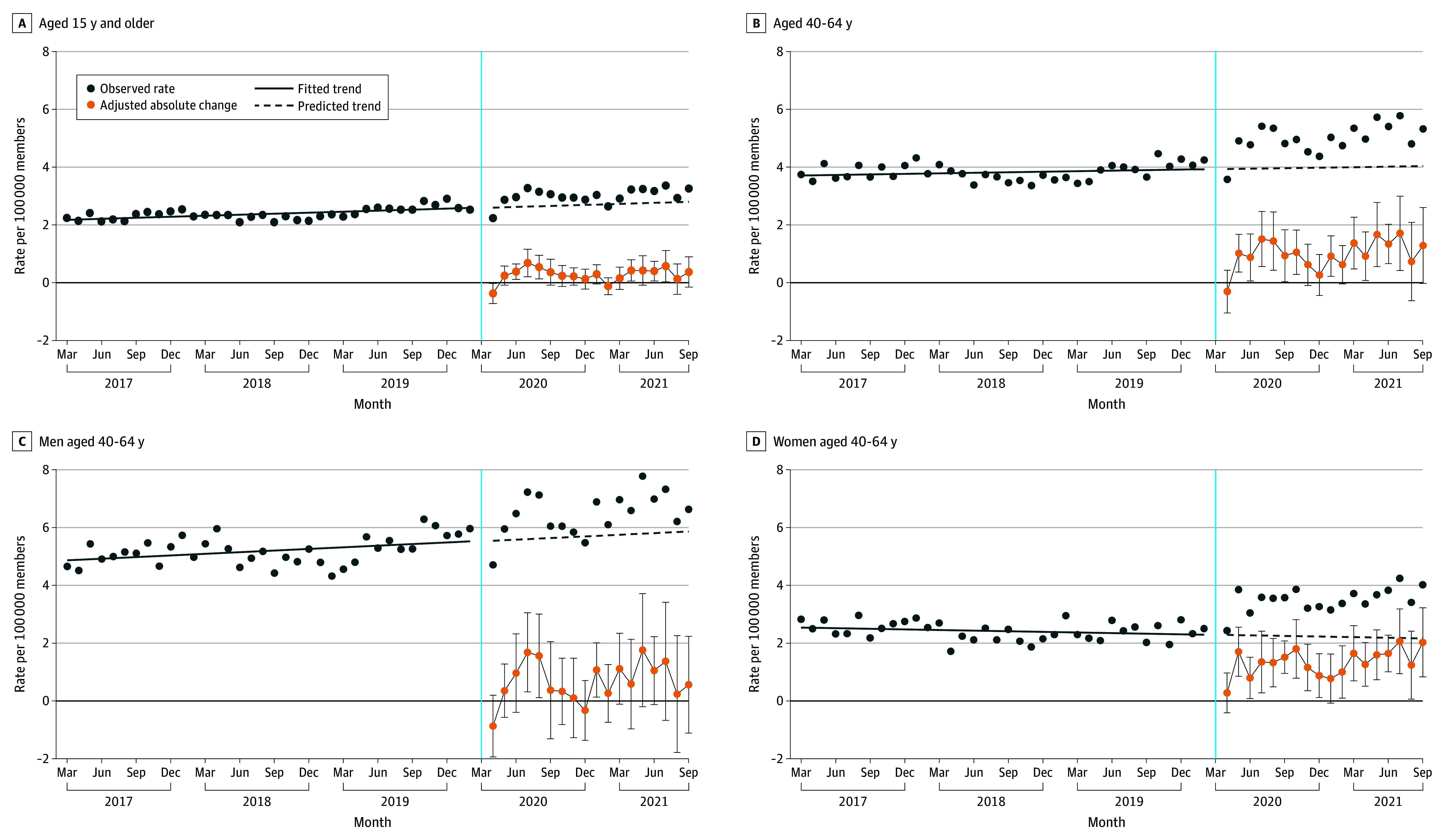
Monthly Rates of High-Acuity Alcohol-Related Liver Disease Complication Episodes Among a National Commercially Insured Population Fitted and predicted rates were generated using unadjusted segmented regression. Absolute change estimates and 95% CIs (error bars) were generated from regression model coefficients using nonlinear combinations of parameters adjusted for member-level age category, sex, US division, seasonality, and poverty level of residence based on 5-digit zip code. A, Complication episodes across all age groups comprise alcohol-related liver disease diagnoses representing acute decompensation of chronic alcohol-related diseases presenting to the emergency department, observation unit, or hospital. Diagnoses were independently developed and cross-verified by 2 authors (B.S. and J.F.W.). There was 95% agreement with classifications, and differences were reviewed and reconciled. B, Adults aged 40 to 64 years are shown because this age category had high rates of alcohol-related morbidity and mortality. Age category was not included in the regression model for analyses depicted. C and D, Male and female adults aged 40 to 64 years are shown to highlight differences in high-acuity alcohol-related complications. Age category and sex were not included in the regression models for analyses depicted. The vertical blue line indicates the time of declaration of the COVID-19 public health emergency.

The overall pattern of high-acuity ALD-related episode rates was similar to that of the broad complication outcome among the overall sample, the subgroups of members aged 40 to 64 years, and women and men in this age group (Figure 2). However, women aged 40 to 64 years experienced statistically significant increases in 16 of 18 pandemic months, with a range of absolute and relative increases of 0.8 to 2.1 episodes per 100 000 people and 34.1% to 94.7%, respectively. Additional subgroup analyses of high-acuity ALD-related episodes by age group and sex revealed no consistent pandemic-era patterns among subgroups and few statistically significant changes (eTable 5 in Supplement 1).

## Discussion

The COVID-19 pandemic was associated with increases in high-acuity alcohol-related complication episodes in half of follow-up months among members aged 40 to 64 years in a national, commercially insured population. Women in this age group experienced increases in 10 of 18 follow-up months, which appeared to be associated with major relative increases in high-acuity ALD-related presentations in 16 of 18 pandemic months.

The increase in ALD-related complication episodes that we detected is consistent with recent analyses that have demonstrated increases in ALD-related hospitalizations and deaths, as well as alcohol-related deaths during the pandemic era.^2,6,7,8,9,10^ Moreover, we add the key findings that the pandemic was associated with increases in a broader measure of alcohol-related complications and that changes primarily occurred among women aged 40 to 64 years. Rates of alcohol-related complications in this group remained elevated into late 2021, suggesting potentially sustained increases in alcohol-related harms. Although estimates among men aged 40 to 64 years did not reach statistical significance, they were consistently higher than predicted after April 2020, warranting further investigation with additional data sources.

Recent epidemiological patterns in alcohol use might help explain these results. Over the past decade, the 2-week prevalence of having 5 or more drinks in a row increased twice as quickly among women aged 35 to 50 years compared with men.^1^ Unlike men, women had considerably more heavy drinking days (≥4 drinks within a few hours) in 2020 compared with 2019.^5^ Thus, longer-term increases in alcohol consumption might have increased the risk of ALD among women aged 40 to 64 years prior to the pandemic, then pandemic-related increases in alcohol consumption may have contributed to new or worsening ALD complications.

These findings imply a need for increased attention to alcohol use disorder (AUD) risk factors, alcohol use patterns, alcohol-related health effects, and related interventions, especially among women aged 40 to 64 years. Clinicians and population health managers should consider increasing screening efforts in this population. Policymakers should consider enhancing access to interventions including AUD treatment early in the disease process and closer collaboration with addiction clinicians and hepatologists. Evidence-based policies to reduce the aforementioned harms include increasing alcohol taxes, setting minimum alcohol prices, and limiting alcohol advertising.^11^ The persistent elevations in alcohol-related complications we detected in a relatively small population subgroup suggest a need for more granular public health monitoring. This study adds a novel, broad measure of acute decompensation of chronic alcohol-related diseases that might reflect alcohol consumption levels in the community, access to AUD treatment, and access to specialists such as hepatologists. Monitoring this measure could complement monitoring less common outcomes such as alcohol-related mortality, allowing identification of smaller, at-risk patient subgroups that require urgent and early interventions.

### Limitations

Study limitations include underestimating high-acuity alcohol-related complications due to use of alcohol-specific billing codes, and potential changes in insurance enrollment patterns from before to after the pandemic. Further research is needed to determine whether the increases we observed persist several years after the pandemic onset.

## Conclusions

In this cohort study, high-acuity alcohol-related complications, especially those associated with ALD, increased during the COVID-19 pandemic period among adults aged 40 to 64 years, particularly women. Findings underscore the need for increased attention to alcohol use disorder risk factors, alcohol use patterns, alcohol-related health effects, and alcohol regulations and policies, especially among women aged 40 to 64 years.

## Notes

Supplement 1.
eAppendix.
**eTable 1.** ICD-10 diagnostic billing codes used to identify high-acuity alcohol-related complications **eTable 2.** Population characteristics (n, %) in April 2017, April 2020, and April 2021 **eTable 3.** Diagnostic clusters of high-acuity alcohol-related complications among a national commercially insured population aged 15 years and older presenting to the emergency department, observation unit, and hospital during the pre- and post-pandemic period **eTable 4.** Absolute and relative changes in monthly rates of high-acuity alcohol-related complication episodes versus predicted rates during the April 2020 to September 2021 pandemic era **eTable 5.** Absolute and relative changes in monthly rates of high-acuity alcohol-related liver disease complication episodes versus predicted rates during the April 2020 to September 2021 pandemic era

Supplement 2. Data Sharing Statement
