# Supplementary material for: High-Acuity Alcohol-Related Complications During the COVID-19 Pandemic
Source: JAMA Health Forum. 2024 Apr 12;5(4):e240501. doi: 10.1001/jamahealthforum.2024.0501 (PMC11065164; doi:10.1001/jamahealthforum.2024.0501)
Supplement: Supplement 1. — eAppendix. eTable 1. ICD-10 diagnostic billing codes used to identify high-acuity alcohol-related complications eTable 2. Population characteristics (n, %) in April 2017, April 2020, and April 2021 eTable 3. Diagnostic clusters of high-acuity alcohol-related complications among a national commercially insured population aged 15 years and older presenting to the emergency department, observation unit, and hospital during the pre- and post-pandemic period eTable 4. Absolute and relative changes in monthly rates of high-acuity alcohol-related complication episodes versus predicted rates during the April 2020 to September 2021 pandemic era eTable 5. Absolute and relative changes in monthly rates of high-acuity alcohol-related liver disease complication episodes versus predicted rates during the April 2020 to September 2021 pandemic era [file jamahealthforum-e240501-s001.pdf]

Supplemental Online Content

Shuey B, Halbisen A, Lakoma M, et al. High-acuity alcohol-related complications during the COVID-19 pandemic. *JAMA Health Forum*. 2024;5(4):e240501. doi:10.1001/jamahealthforum.2024.0501

eAppendix.

eTable 1. ICD-10 diagnostic billing codes used to identify high-acuity alcohol-related complications

eTable 2. Population characteristics (n, %) in April 2017, April 2020, and April 2021

eTable 3. Diagnostic clusters of high-acuity alcohol-related complications among a national commercially insured population aged 15 years and older presenting to the emergency department, observation unit, and hospital during the pre- and post-pandemic period

eTable 4. Absolute and relative changes in monthly rates of high-acuity alcohol-related complication episodes versus predicted rates during the April 2020 to September 2021 pandemic era

eTable 5. Absolute and relative changes in monthly rates of high-acuity alcohol-related liver disease complication episodes versus predicted rates during the April 2020 to September 2021 pandemic era

This supplemental material has been provided by the authors to give readers additional information about their work.

© 2024 Shuey B et al. *JAMA Health Forum.*

eAppendix

I. eMethods

A. Creation of high-acuity alcohol-related complication episodes

We created high-acuity alcohol-related complication episodes by generating 10-day windows (beginning with the day of a high-acuity alcohol-related visit to the emergency department, observation unit, or hospital) during which we captured visits with high-acuity alcohol-related diagnoses (eTable 1). This approach prevented "double counting" of situations such as a 3-day hospitalization for alcohol-related cirrhosis with ascites followed by a related emergency department visit 2 days later. We required that the diagnosis of interest be in the first through fifth position on the claim. Over the 10-day window, we included only encounters in the emergency department, observation unit, or hospital 10-day episodes because these are likely to represent higher acuity presentations (in contrast to outpatient- or telehealth-based care). Our measure of interest was the number of high-acuity episodes per member per month presenting to the health system.

We generated diagnostic clusters at each encounter level (highest of observation unit, emergency department, or inpatient unit) in the pre- and post-pandemic periods to assess changes in diagnoses driving episodes of high-acuity alcohol-related complications across the study period. We present the total number of diagnostic clusters at each encounter level in the pre- and post-pandemic periods in eTable 3.

B. Statistical analysis

We hypothesized that high-acuity alcohol-related complications would initially decrease in the early months of the pandemic compared to levels predicted by the pre-pandemic trend, then later increase above predicted. We did not have an *a priori* hypothesis regarding the timing of a transition from decreases to increases versus expected because of uncertainty regarding the timing of complication presentations after a rapid increase in population-level alcohol use and uncertainty about how long complication presentations might be delayed in the COVID environment that reduced access to care and had extensive stay-at-home and social distancing measures. Adults aged 40-64 years have higher rates of alcohol-related liver disease and alcohol-related mortality rates than other age groups.[1,2] Therefore, we hypothesized that this age group would have higher rates of high-acuity alcohol-related complications compared to other age groups. The annual percentage change in alcohol-related age-adjusted mortality rates has increased faster among women compared to men during the last decade.[2] Additionally, women consumed significantly more alcohol and binge drank more during 2020 compared to 2019 while men did not.[3] Therefore, we hypothesized that women would have higher rates of high-acuity alcohol-related complications compared to men.

Data were organized at the person-month level. March 2017, the beginning of the study period, was labeled month 1, and September 2021, the end of the study period, was labeled month 54. We considered March 2020 a phase-in month given that the first Stay-At-Home orders were declared during this month and excluded March 2020 observations from the analysis. We modeled a linear term for the baseline trend in high-acuity alcohol-related complications because previous literature has demonstrated linear trends in alcohol-related harms and because we had no reason to suspect it would be non-linear.[4,5] However, because we were uncertain about the functional form of this trend during the pandemic, we conservatively modeled the post-March 2020 period using indicators for each follow-up month rather than e.g. a linear or quadratic trend:

$$\boldsymbol{y_{it} = B_0 + B_1 PreTrend_{it} + B_2 Post_{,i1} + B_3 Post_{i2} + \cdots B_{19} Post_{i18} + B_{20} X_{it} + B_{21} SeasonalQuarter_{1it} + B_{22} SeasonalQuarter_{2it} + B_{23} SeasonalQuarter_{3it} + \varepsilon_{it}}$$

Where:

$\boldsymbol{y_{it}}$ is the dependent variable equal to the monthly rate per 100,000 members of high-acuity alcohol-related complications for individual *i* at month *t*,

$\boldsymbol{PreTrend_i}$ is the baseline continuous trend term which starts at 1 in March 2017, counts upward to 36 (February 2020, the final baseline month), and is otherwise equal to 0 for the remaining months of the study period,

© 2024 Shuey B et al. *JAMA Health Forum.*

Post$_{1,2,...,18}$ refer to unique indicators for each follow-up month (*Post*$_{i1}$ is an indicator equal to 1 during the first follow-up month (April 2020) and equal to 0 during all other months, *Post*$_{i2}$ is an indicator equal to 1 during the second follow-up month (May 2020) and equal to 0 during all other months,… *Post*$_{i18}$ is an indicator equal to 1 during the 18th and final follow-up month (September 2021) and equal to 0 during all other months)
$\boldsymbol{X_{it}}$ is equal to a vector of covariates for individual *i* at month *t,*
SeasonalQuarter$_{1,2,3i}$ is an indicator for quarterly seasons where *SeasonalQuarter*$_1$ is equal to 1 during months March, April, and May, and equal to 0 in other months; *SeasonalQuarter*$_2$ is equal to 1 during months June, July, and August, and equal to 0 in other months; and *SeasonalQuarter*$_3$ is equal to 1 during months September, October, and November, and equal to 0 in other months. We chose *SeasonalQuarter*$_4$ (December, January, and February) as the reference group given that this was the last quarter before the Covid-19 pandemic and because winter has the lowest rates of alcohol use of the seasons.[6]
$\boldsymbol{\varepsilon_{it}}$ is equal to the error term for individual *i* at month *t,*
$\boldsymbol{B_0}$ is equal to a constant coefficient for the intercept,
$\boldsymbol{B_1}$ is the coefficient for the baseline trend term,
$\boldsymbol{B_{2\text{-}19}}$ are the coefficients for indicators for each of the 18 follow-up months,
$\boldsymbol{B_{20}}$ is equal to the coefficient vector for the vector of covariates
$\boldsymbol{B_{21\text{-}23}}$ are the coefficients for quarterly seasonal indicators 1 (March, April, May), 2 (June, July, August), and 3 (September, October, November).

Absolute and relative changes at each follow-up month

We used the above modeling approach to estimate the adjusted absolute rate change in observed high-acuity alcohol-related complications compared to predicted at each follow-up month. We generated person-level generalized estimating equations using an identity link with robust sandwich estimators clustering at the member ID level. We then used non-linear combination of parameters to calculate the **absolute change** with 95% confidence intervals in each pandemic month's adjusted rate from the pandemic month's predicted rate extended from the baseline linear trend (e.g., the adjusted observed rate in April 2020 compared to the predicted rate in April 2020). Relative changes using adjusted models are sensitive to the referent group chosen for each covariate in each model and may provide inaccurate relative change estimates. Therefore, we used unadjusted models to calculate the **relative change** by dividing the unadjusted absolute change (unadjusted rate minus unadjusted predicted rate) by the unadjusted predicted rate for each pandemic month.

Unadjusted plots

We ran identical models as described above but unadjusted for X_{it} (a vector of covariates for individual *i* at month *t)* to generate plots of observed monthly values, the observed fitted linear trend, and a predicted linear trend extended into the pandemic period.

References

1. Choi G, Runyon BA. Alcoholic Hepatitis: A Clinician's Guide. *Clinics in Liver Disease*. 2012;16(2):371-385. doi:10.1016/j.cld.2012.03.015

2. Karaye IM, Maleki N, Hassan N, Yunusa I. Trends in Alcohol-Related Deaths by Sex in the US, 1999-2020. *JAMA Network Open*. 2023;6(7):e2326346. doi:10.1001/jamanetworkopen.2023.26346

3. Pollard MS, Tucker JS, Green HD. Changes in Adult Alcohol Use and Consequences During the COVID-19 Pandemic in the US. *JAMA Netw Open*. 2020;3(9):e2022942. doi:10.1001/jamanetworkopen.2020.22942

4. Spencer M, Curtin S, Garnett M. *Alcohol-Induced Death Rates in the United States, 2019–2020.* National Center for Health Statistics; 2022. Accessed January 2, 2024. https://www.cdc.gov/nchs/products/databriefs/db448.htm#ref2

© 2024 Shuey B et al. *JAMA Health Forum.*

5. Sohal A, Chaudhry H, Patel J, et al. Impact of COVID-19 pandemic on alcohol-related hepatitis admissions: Analysis of nationwide data 2016-2020. *Am J Med Sci*. Published online June 12, 2023. doi:10.1016/j.amjms.2023.06.002

6. Olson KL, Whitley P, Velasco J, LaRue L, Dawson E, Huskey A. Seasonal and Regional Influences on Alcohol Consumption: An Analysis of Near-Real-Time Urine Drug Test Results in Those Seeking Health Care. *Drug and Alcohol Dependence*. 2021;227:108908. doi:10.1016/j.drugalcdep.2021.108908

eTable 1. ICD-10 diagnostic billing codes used to identify high-acuity alcohol-related complications

Code	Diagnosis
F10121	Alcohol abuse with intoxication delirium
F10131	Alcohol abuse with withdrawal delirium
F10132	Alcohol abuse with withdrawal perceptual disturbances
F1015	Alcohol abuse with induced psychotic disorder
F10150	Alcohol abuse with induced psychotic disorder with delusions
F10151	Alcohol abuse with induced psychotic disorder with hallucinations
F10159	Alcohol abuse with induced psychotic disorder, unspecified
F10221	Alcohol dependence with intoxication delirium
F10231	Alcohol dependence with withdrawal delirium
F10232	Alcohol dependence with withdrawal perceptual disturbances
F1025	Alcohol dependence with induced psychotic disorder
F10250	Alcohol dependence with induced psychotic disorder with delusions
F10251	Alcohol dependence with induced psychotic disorder with hallucinations
F10259	Alcohol dependence with induced psychotic disorder, unspecified
F10921	Alcohol use, unspecified, with intoxication delirium
F10931	Alcohol use, unspecified, with withdrawal delirium
F10932	Alcohol use, unspecified, with withdrawal with perceptual disturbances
F1095	Alcohol use, unspecified, with induced psychotic disorder
F10950	Alcohol use, unspecified, with induced psychotic disorder with delusions
F10951	Alcohol use, unspecified, with induced psychotic disorder with hallucinations
F10959	Alcohol use, unspecified, with induced psychotic disorder, unspecified
I426	Alcohol-related cardiomyopathy
K2921	Alcohol-related gastritis with bleeding
K7010	Alcohol-related hepatitis without ascites
K7011	Alcohol-related hepatitis with ascites
K7031	Alcohol-related cirrhosis of the liver with ascites
K7040	Alcohol-related hepatic failure without coma
K7041	Alcohol-related hepatic failure with coma
K852	Alcohol induced acute pancreatitis

© 2024 Shuey B et al. *JAMA Health Forum.*

eTable 2. Population characteristics (n, %) in April 2017, April 2020, and April 2021

	**April 2017**		**April 2020**		**April 2021**	
Population size, n	14,349,252		14,735,764		14,672,902	
Female	7,420,018	(51.7%)	7,691,573	(52.2%)	7,727,257	(52.7%)
Age at index date, years						
15-39	4,660,728	(32.5%)	4,255,239	(28.9%)	3,887,882	(26.5%)
40-64	5,073,821	(35.4%)	4,724,252	(32.1%)	4,465,405	(30.4%)
65-74	2,529,202	(17.6%)	3,115,081	(21.1%)	3,407,471	(23.2%)
75+	2,085,501	(14.5%)	2,641,192	(17.9%)	2,912,144	(19.9%)
US Division[2]						
New England	397,023	(2.8%)	544,979	(3.7%)	588,814	(4.0%)
Mid-Atlantic	1,073,664	(7.5%)	1,052,602	(7.1%)	1,046,067	(7.1%)
East North Central	1,981,814	(13.8%)	2,029,957	(13.8%)	2,077,220	(14.2%)
West North Central	1,222,024	(8.5%)	1,366,349	(9.3%)	1,408,948	(9.6%)
South Atlantic	3,200,251	(22.3%)	3,390,032	(23.0%)	3,239,049	(22.1%)
East South Central	590,334	(4.1%)	600,967	(4.1%)	647,970	(4.4%)
West South Central	2,383,998	(16.6%)	2,039,284	(13.8%)	2,204,779	(15.0%)
Mountain	1,365,729	(9.5%)	1,517,566	(10.3%)	1,569,907	(10.7%)
Pacific	1,814,238	(12.6%)	1,818,190	(12.3%)	1,828,344	(12.5%)
Missing	320,177	(2.2%)	375,838	(2.6%)	61,804	(0.4%)
Poverty level of residence						
Living in lower-income zip code[1]	4,395,777	(30.6%)	4,441,199	(30.1%)	4,494,480	(30.6%)

[1]Defined as >=15% of households in the zip code living below the federal poverty level. [2]People with missing state of residence were included in analyses.

© 2024 Shuey B et al. *JAMA Health Forum.*

eTable 3. Diagnostic clusters of high-acuity alcohol-related complications among a national commercially insured population aged 15 years and older presenting to the emergency department, observation unit, and hospital during the pre- and post-pandemic period

	**Diagnostic clusters, n (%) [a]**					
	**Pre-pandemic (March 2017-February 2020)**			**Post-pandemic (April 2020-September 2021)**		
	ED	Observation Unit	Inpatient Unit	ED	Observation Unit	Inpatient Unit
1. High-acuity alcohol-related liver disease [b]	9929 (58.2%)	3775 (65.1%)	11879 (54.2%)	6609 (63.7%)	2357 (66.3%)	6885 (57.4%)
2. High-acuity alcohol withdrawal or alcohol-related mood disorder [c]	6175 (36.2%)	1697 (29.3%)	8568 (39.1%)	3266 (31.5%)	1036 (29.1%)	4403 (36.8%)
3. Alcohol-related cardiomyopathy [d]	538 (3.2%)	247 (4.3%)	1178 (5.4%)	277 (2.7%)	112 (3.2%)	531 (4.4%)
4. Alcohol-related gastritis with bleeding [e]	428 (2.5%)	82 (1.4%)	304 (1.4%)	215 (2.1%)	52 (1.5%)	172 (1.4%)

Abbreviation: ED, emergency department.

[a] The total number in each row represents unique diagnostic codes aggregated into diagnostic clusters during the pre-pandemic and post-pandemic periods (note that total number of diagnostic clusters are higher in the pre-pandemic period given the longer pre-pandemic window compared to the post-pandemic window). These diagnostic codes were de-duplicated at the daily level such that, for example, a member with five ICD-10 codes for *alcohol-related cirrhosis with ascites* on day 1 of hospitalization would only contribute 1 code to the diagnostic cluster "high-acuity alcohol-related liver disease." A member admitted to the hospital for 5 days with an ICD-10 code for *alcohol-related cirrhosis with ascites* on each hospital day would contribute 5 codes to the diagnostic cluster "high-acuity alcohol-related liver disease".

[b] High-acuity alcohol-related liver disease includes any liver-related ICD-10 code from eTable 1 (K7010, K7011, K7031, K7040, K7041).

[c] High-acuity alcohol withdrawal or alcohol-related mood disorder includes any alcohol withdrawal delirium or alcohol-related mood disorder ICD-10 codes from eTable 1 (F10121, F10131, F10132, F1015, F10150, F10151, F10159, F10221, F10231, F10232, F1025, F10250, F10251, F10259, F10921, F10931, F10932, F1095, F10950, F10951, F10959).

[d] Alcohol-related cardiomyopathy includes one ICD-10 code from eTable 1 (I426).

[e] Alcohol-related gastritis with bleeding includes one ICD-10 code from eTable 1 (K2921).

© 2024 Shuey B et al. *JAMA Health Forum.*

eTable 4. Absolute and relative changes in monthly rates of high-acuity alcohol-related complication episodes versus predicted rates during the April 2020 to September 2021 pandemic era

1. 15-Year-Old and Older Subgroup (Full Sample)

Month[a]	Change in composite high-acuity alcohol-related complication episodes per 100,000 people					
	Aged 15+		Aged 15+, Males		Aged 15+, Females	
	Absolute[b] (95% CI)	Relative[c] %	Absolute (95% CI)	Relative %	Absolute (95% CI)	Relative %
Apr 2020	-0.7 (-1.2, -0.1)*	-13.6	-1.4 (-2.3, -0.6)**	-17.9	0 (-0.4, 0.4)	-2.2
May 2020	0.4 (-0.2, 0.9)	9	0 (-0.7, 0.7)	2.9	0.7 (0.2, 1.2)**	24.9
Jun 2020	0.4 (0, 0.8)*	8.3	0.8 (0, 1.6)*	12.9	0 (-0.4, 0.4)	-2.4
Jul 2020	0.7 (-0.1, 1.5)	15.7	0.8 (-0.2, 1.9)	13.6	0.6 (0, 1.3)	21.8
Aug 2020	0.5 (0, 1)	11.3	0.5 (-0.4, 1.3)	8.6	0.6 (0.1, 1)*	18.7
Sep 2020	0.6 (0, 1.1)*	14.5	0.4 (-0.6, 1.4)	8	0.7 (0.3, 1.2)***	31.7
Oct 2020	0.3 (-0.2, 0.8)	9	0.2 (-0.6, 0.9)	5	0.4 (-0.1, 1)	20
Nov 2020	0.1 (-0.4, 0.6)	4.9	0 (-0.8, 0.7)	2.7	0.2 (-0.2, 0.6)	11.1
Dec 2020	0.1 (-0.3, 0.5)	2.9	0 (-0.8, 0.7)	0.2	0.2 (-0.2, 0.7)	10.5
Jan 2021	0.3 (-0.2, 0.9)	7.2	0.2 (-0.6, 0.9)	3	0.5 (0, 1)*	18.6
Feb 2021	-0.3 (-0.7, 0.1)	-6.3	-0.5 (-1.2, 0.3)	-6	-0.2 (-0.6, 0.3)	-6.2
Mar 2021	0.2 (-0.5, 0.8)	5.1	-0.2 (-1.1, 0.7)	1.3	0.5 (-0.1, 1)	15.5
Apr 2021	0.6 (-0.1, 1.2)	14.7	0.4 (-0.5, 1.4)	11.5	0.7 (0.1, 1.3)*	24.5
May 2021	0.7 (0, 1.4)*	17.7	0.9 (-0.3, 2.1)	18.8	0.5 (0, 1)*	17.2
Jun 2021	0.5 (0, 1.1)	12.8	0.3 (-0.6, 1.2)	8.9	0.7 (0.2, 1.2)**	24.5
Jul 2021	0.8 (0.1, 1.6)*	19.4	0.8 (-0.4, 1.9)	15.6	0.9 (0.3, 1.4)**	31
Aug 2021	0.1 (-0.6, 0.8)	4.1	-0.1 (-1.3, 1.1)	3	0.3 (-0.2, 0.8)	8.9
Sep 2021	0.3 (-0.5, 1.1)	10.1	0.1 (-1.1, 1.2)	6.3	0.5 (-0.1, 1.1)	21.6

*p<0.05, **p<0.01, ***p<0.001

[a] Table displays absolute and relative changes (versus predicted rates) in monthly rates of high-acuity alcohol-related complication episodes presenting to the emergency department, observation unit, or hospital.
[b] Absolute change estimates and 95% confidence intervals generated from adjusted regression model coefficients using nonlinear combinations of parameters.
[c] Relative change estimates calculated by dividing the unadjusted absolute change (unadjusted observed rate minus unadjusted predicted rate) by the unadjusted predicted rate for each pandemic month. Given that the adjusted relative changes are sensitive to the referent group chosen for each covariate in each model, we elected to calculate unadjusted relative changes.
[d] Models adjusted for member-level age category, sex, US division, seasonality, and poverty level of residence based on 5-digit zip code. Age category not included in models stratified by age and sex not included in models stratified by sex.

2. 15-39-Year-Old Subgroup

Month[a]	Change in composite high-acuity alcohol-related complication episodes per 100,000 people					
	Aged 15-39		Aged 15-39, Males		Aged 15-39, Females	
	Absolute[b] (95% CI)	Relative[c] %	Absolute (95% CI)	Relative %	Absolute (95% CI)	Relative %
Apr 2020	-0.2 (-0.6, 0.1)	-14.7	-0.7 (-1.4, 0)*	-27.5	0.3 (-0.2, 0.7)	14.6
May 2020	0.2 (-0.3, 0.6)	5.8	0.3 (-0.5, 1.1)	8.7	0.1 (-0.3, 0.4)	-0.3
Jun 2020	0.1 (-0.4, 0.5)	-0.1	0.2 (-0.5, 1)	8.5	-0.1 (-0.6, 0.4)	-19
Jul 2020	0.3 (-0.2, 0.8)	9.6	0.5 (-0.3, 1.3)	17.4	0 (-0.5, 0.6)	-7.4
Aug 2020	0.6 (0.2, 1.1)**	26.8	0.7 (0, 1.4)	24.2	0.6 (0.1, 1.1)*	33.4
Sep 2020	0.1 (-0.5, 0.7)	12.3	-0.2 (-1.3, 0.8)	-2	0.5 (-0.2, 1.1)	45
Oct 2020	0 (-0.5, 0.4)	5.5	-0.1 (-0.8, 0.7)	3	0 (-0.5, 0.6)	11.7
Nov 2020	-0.4 (-0.9, 0.1)	-12	-0.5 (-1.1, 0.2)	-10.6	-0.3 (-0.9, 0.2)	-14.7
Dec 2020	0.1 (-0.5, 0.6)	1.5	0 (-0.9, 1)	-0.8	0.1 (-0.4, 0.7)	7.3
Jan 2021	0.6 (0.1, 1.1)*	27.1	0.2 (-0.7, 1.1)	5.3	1.1 (0.4, 1.8)**	76.5
Feb 2021	-0.3 (-0.8, 0.2)	-14.9	-0.5 (-1.5, 0.4)	-20.1	0 (-0.6, 0.6)	-2.8
Mar 2021	0.2 (-0.5, 0.8)	5	0.2 (-0.7, 1.1)	5.7	0.1 (-0.5, 0.8)	3.9
Apr 2021	0.4 (-0.1, 0.9)	19.1	0.4 (-0.6, 1.4)	15.6	0.4 (-0.1, 1)	28.2
May 2021	0.2 (-0.3, 0.7)	8	0.2 (-0.5, 1)	10.6	0.1 (-0.5, 0.7)	3.5
Jun 2021	0.4 (-0.3, 1)	17.3	0.2 (-0.6, 1)	11	0.6 (-0.2, 1.3)	32.8
Jul 2021	0.3 (-0.3, 0.8)	11.8	0 (-0.8, 0.9)	4.9	0.5 (-0.1, 1.1)	28.5
Aug 2021	0.1 (-0.5, 0.6)	3	-0.4 (-1.2, 0.4)	-9.3	0.6 (-0.1, 1.2)	31.6
Sep 2021	0 (-0.5, 0.6)	9.8	0.1 (-0.9, 1)	10.2	0 (-0.7, 0.6)	10

© 2024 Shuey B et al. *JAMA Health Forum.*

*p<0.05, **p<0.01, ***p<0.001

[a] Table displays absolute and relative changes (versus predicted rates) in monthly rates of high-acuity alcohol-related complication episodes presenting to the emergency department, observation unit, or hospital.
[b] Absolute change estimates and 95% confidence intervals generated from adjusted regression model coefficients using nonlinear combinations of parameters.
[c] Relative change estimates calculated by dividing the unadjusted absolute change (unadjusted observed rate minus unadjusted predicted rate) by the unadjusted predicted rate for each pandemic month. Given that the adjusted relative changes are sensitive to the referent group chosen for each covariate in each model, we elected to calculate unadjusted relative changes.
[d] Models adjusted for member-level age category, sex, US division, seasonality, and poverty level of residence based on 5-digit zip code. Age category not included in models stratified by age and sex not included in models stratified by sex.

3. 40–64-Year-Old Subgroup

Month[a]	Change in composite high-acuity alcohol-related complication episodes per 100,000 people					
	Aged 40-64		Aged 40-64, Males		Aged 40-64, Females	
	Absolute[b] (95% CI)	Relative[c] %	Absolute (95% CI)	Relative %	Absolute (95% CI)	Relative %
Apr 2020	-0.6 (-1.8, 0.6)	-6.3	-1.4 (-3.2, 0.4)	-11.6	0.3 (-0.7, 1.3)	8.1
May 2020	1.3 (0.2, 2.5)*	22.2	0.9 (-0.8, 2.6)	12.9	1.8 (0.7, 2.8)**	47.3
Jun 2020	1.1 (0, 2.1)*	16.9	1.6 (-0.3, 3.5)	19.2	0.5 (-0.4, 1.4)	12.2
Jul 2020	1.5 (-0.1, 3)	23.1	1.6 (-0.5, 3.8)	19.5	1.3 (-0.1, 2.7)	33.9
Aug 2020	0.9 (-0.4, 2.1)	14	0.4 (-1.5, 2.3)	7.2	1.3 (0.3, 2.3)*	33.3
Sep 2020	1.1 (0.1, 2.1)*	17.1	0.6 (-1.2, 2.4)	8	1.7 (0.8, 2.5)***	42.3
Oct 2020	1.2 (0.3, 2.1)**	18.9	1.1 (-0.4, 2.5)	13.4	1.4 (0.3, 2.4)**	35
Nov 2020	0.2 (-0.9, 1.4)	4.7	-0.6 (-2.3, 1.2)	-3.3	1.1 (-0.1, 2.3)	27.1
Dec 2020	0.5 (-0.3, 1.4)	8.2	0.4 (-1.1, 1.8)	3.2	0.7 (-0.1, 1.6)	22.9
Jan 2021	1.2 (0, 2.3)*	17.5	1.3 (-0.5, 3.1)	12.6	1.1 (0.2, 2)*	32.1
Feb 2021	0.6 (-0.5, 1.7)	8.9	0.4 (-1.4, 2.1)	3.3	0.8 (-0.1, 1.8)	25
Mar 2021	1.4 (0.1, 2.7)*	23.7	0.8 (-1.1, 2.8)	13.1	2 (0.8, 3.1)**	53.3
Apr 2021	0.9 (-0.6, 2.4)	18.4	0.6 (-1.6, 2.7)	12.7	1.3 (-0.1, 2.6)	35.9
May 2021	2.1 (0.4, 3.8)*	35.9	2.6 (-0.3, 5.5)	33	1.6 (0.5, 2.8)**	46.5
Jun 2021	1.6 (0.5, 2.6)**	26.7	1.2 (-0.6, 3)	18.1	1.9 (1, 2.9)***	52.7
Jul 2021	2.1 (0.4, 3.7)*	34	2.1 (-0.6, 4.7)	27	2.1 (0.8, 3.4)**	56
Aug 2021	0.6 (-1.1, 2.4)	13.5	0.4 (-2.2, 3)	10.1	0.9 (-0.5, 2.3)	25.4
Sep 2021	1 (-0.9, 2.9)	17.5	0.3 (-2.4, 3)	8.6	1.6 (0.2, 3.1)*	44

*p<0.05, **p<0.01, ***p<0.001

[a] Table displays absolute and relative changes (versus predicted rates) in monthly rates of high-acuity alcohol-related complication episodes presenting to the emergency department, observation unit, or hospital.
[b] Absolute change estimates and 95% confidence intervals generated from adjusted regression model coefficients using nonlinear combinations of parameters.
[c] Relative change estimates calculated by dividing the unadjusted absolute change (unadjusted observed rate minus unadjusted predicted rate) by the unadjusted predicted rate for each pandemic month. Given that the adjusted relative changes are sensitive to the referent group chosen for each covariate in each model, we elected to calculate unadjusted relative changes.
[d] Models adjusted for member-level age category, sex, US division, seasonality, and poverty level of residence based on 5-digit zip code. Age category not included in models stratified by age and sex not included in models stratified by sex.

4. 65-74-Year-Old Subgroup

Month[a]	Change in composite high-acuity alcohol-related complication episodes per 100,000 people					
	Aged 65-74		Aged 65-74Males		Aged 65-74Females	
	Absolute[b] (95% CI)	Relative[c] %	Absolute (95% CI)	Relative %	Absolute (95% CI)	Relative %
Apr 2020	-1.3 (-2.4, -0.2)*	-19.5	-2.7 (-4.4, -0.9)**	-22.4	-0.2 (-1.2, 0.9)	-13.2
May 2020	-0.6 (-1.8, 0.6)	-8.8	-1.2 (-2.9, 0.4)	-8.7	0 (-1.2, 1.1)	-9.4
Jun 2020	0.3 (-0.5, 1.2)	4.2	1.4 (-0.4, 3.1)	13.5	-0.5 (-1.1, 0.2)	-18.5
Jul 2020	1.1 (-0.3, 2.5)	15.7	1.4 (-0.7, 3.5)	13.8	0.9 (-0.3, 2.1)	19.7
Aug 2020	0.5 (-0.6, 1.5)	6	1.2 (-0.4, 2.7)	11.9	-0.1 (-1.2, 1)	-8.1
Sep 2020	0.8 (-0.4, 2)	13.1	1.7 (-0.6, 4.1)	14.6	0.1 (-1.2, 1.3)	9.3
Oct 2020	-0.2 (-1.4, 0.9)	-2.4	0 (-2.2, 2.3)	-1.1	-0.5 (-1.6, 0.6)	-5.6
Nov 2020	0.3 (-0.6, 1.2)	5.4	1.2 (-0.5, 2.9)	9.3	-0.4 (-1.6, 0.8)	-3.7

© 2024 Shuey B et al. *JAMA Health Forum.*

Dec 2020	-0.6 (-1.7, 0.4)	-9.8	-1 (-2.9, 1)	-8.1	-0.4 (-1.3, 0.5)	-13.7
Jan 2021	-0.7 (-1.6, 0.3)	-9.9	-0.7 (-2.2, 0.7)	-6.3	-0.6 (-1.5, 0.3)	-19.5
Feb 2021	-1.3 (-2.3, -0.3)*	-19.6	-1.5 (-3.1, 0.2)	-12.8	-1.2 (-2.3, -0.2)*	-36.4
Mar 2021	-0.8 (-2, 0.4)	-12.4	-1.3 (-3.1, 0.4)	-9.4	-0.4 (-1.5, 0.7)	-20.5
Apr 2021	1 (-0.4, 2.3)	13.7	1.1 (-1.1, 3.2)	12.8	0.9 (-0.2, 2)	14.3
May 2021	0.3 (-0.9, 1.5)	3.8	0.7 (-1.5, 3)	9.6	-0.1 (-1.2, 1.1)	-10.6
Jun 2021	-0.1 (-1.4, 1.1)	-3.1	-0.1 (-2.1, 2)	0.4	-0.2 (-1.5, 1)	-12.1
Jul 2021	0.7 (-0.8, 2.2)	8.3	0.6 (-1.9, 3.1)	6.5	0.7 (-0.5, 1.9)	11.4
Aug 2021	-0.4 (-1.5, 0.7)	-6.6	0 (-2, 2)	1	-0.7 (-2, 0.6)	-24.3
Sep 2021	0.4 (-0.8, 1.6)	6.2	1.1 (-1.4, 3.5)	8.4	-0.2 (-1.2, 0.8)	0.5

*p<0.05, **p<0.01, ***p<0.001

[a] Table displays absolute and relative changes (versus predicted rates) in monthly rates of high-acuity alcohol-related complication episodes presenting to the emergency department, observation unit, or hospital.
[b] Absolute change estimates and 95% confidence intervals generated from adjusted regression model coefficients using nonlinear combinations of parameters.
[c] Relative change estimates calculated by dividing the unadjusted absolute change (unadjusted observed rate minus unadjusted predicted rate) by the unadjusted predicted rate for each pandemic month. Given that the adjusted relative changes are sensitive to the referent group chosen for each covariate in each model, we elected to calculate unadjusted relative changes.
[d] Models adjusted for member-level age category, sex, US division, seasonality, and poverty level of residence based on 5-digit zip code. Age category not included in models stratified by age and sex not included in models stratified by sex.

5. 75-Year-Old and Older Subgroup

Month[a]	Change in composite high-acuity alcohol-related complication episodes per 100,000 people					
	Aged 75+		Aged 75+, Males		Aged 75+, Females	
	Absolute[b] (95% CI)	Relative[c] %	Absolute (95% CI)	Relative %	Absolute (95% CI)	Relative %
Apr 2020	-0.9 (-1.6, -0.2)**	-29.8	-1.4 (-2.8, -0.1)*	-25.9	-0.6 (-1, -0.1)*	-40.4
May 2020	0 (-0.9, 0.8)	1.7	-1.2 (-2.2, -0.1)	-19.9	0.7 (-0.1, 1.6)	59.3
Jun 2020	-0.3 (-0.9, 0.4)	-13	-0.6 (-2, 0.8)	-17.8	-0.1 (-0.5, 0.4)	-0.4
Jul 2020	-0.4 (-1, 0.2)	-17	-1 (-2.2, 0.2)	-25.1	0 (-0.5, 0.6)	4.6
Aug 2020	-0.3 (-1, 0.5)	-12.6	-0.8 (-2, 0.5)	-21	0.1 (-0.7, 0.8)	9.7
Sep 2020	0 (-0.4, 0.4)	0	-0.7 (-1.9, 0.5)	-9.3	0.4 (-0.1, 1)	24.9
Oct 2020	-0.2 (-0.8, 0.5)	-5.1	-1.2 (-2.3, -0.2)*	-20.3	0.6 (-0.2, 1.4)	35.1
Nov 2020	0.3 (-0.6, 1.3)	13.1	0.5 (-1.5, 2.5)	14.4	0.2 (-0.5, 1)	10.2
Dec 2020	0.3 (-0.4, 1)	9.4	0.3 (-1, 1.5)	3.6	0.3 (-0.5, 1.1)	25.5
Jan 2021	-0.3 (-0.8, 0.3)	-10.2	-0.8 (-2, 0.4)	-17.8	0.1 (-0.6, 0.8)	9.1
Feb 2021	-0.6 (-1.3, 0.1)	-22.1	-0.7 (-2.2, 0.8)	-16.1	-0.6 (-1, -0.2)**	-39
Mar 2021	-0.7 (-1.2, -0.3)**	-22	-1.5 (-2.5, -0.5)**	-27.4	-0.1 (-0.6, 0.3)	-8.6
Apr 2021	-0.4 (-0.9, 0.1)	-11.1	-0.9 (-2, 0.3)	-13.9	-0.1 (-0.6, 0.4)	-4.3
May 2021	-0.4 (-1, 0.2)	-10.6	-0.9 (-2, 0.2)	-15.1	0 (-0.7, 0.6)	0.4
Jun 2021	-0.2 (-0.8, 0.4)	-9	-0.6 (-1.9, 0.7)	-18	0.1 (-0.4, 0.7)	13.9
Jul 2021	-0.1 (-0.7, 0.4)	-8.4	-0.1 (-1.4, 1.2)	-7.3	-0.2 (-0.7, 0.3)	-12
Aug 2021	-0.2 (-0.8, 0.3)	-11.6	-0.8 (-2.1, 0.5)	-21.9	0.2 (-0.5, 0.9)	14.9
Sep 2021	-0.6 (-1.4, 0.1)	-20.9	-1.7 (-2.9, -0.5)*	-29.9	0.1 (-0.7, 1)	2.1

*p<0.05, **p<0.01, ***p<0.001

[a] Table displays absolute and relative changes (versus predicted rates) in monthly rates of high-acuity alcohol-related complication episodes presenting to the emergency department, observation unit, or hospital.
[b] Absolute change estimates and 95% confidence intervals generated from adjusted regression model coefficients using nonlinear combinations of parameters.
[c] Relative change estimates calculated by dividing the unadjusted absolute change (unadjusted observed rate minus unadjusted predicted rate) by the unadjusted predicted rate for each pandemic month. Given that the adjusted relative changes are sensitive to the referent group chosen for each covariate in each model, we elected to calculate unadjusted relative changes.
[d] Models adjusted for member-level age category, sex, US division, seasonality, and poverty level of residence based on 5-digit zip code. Age category not included in models stratified by age and sex not included in models stratified by sex.

© 2024 Shuey B et al. *JAMA Health Forum.*

eTable 5. Absolute and relative changes in monthly rates of high-acuity alcohol-related liver disease complication episodes versus predicted rates during the April 2020 to September 2021 pandemic era

1. **15-Year-Old and Older Subgroup (Full Sample)**

Month[a]	Change in high-acuity alcohol-related liver disease complication episodes per 100,000 people					
	Overall		Overall, Males		Overall, Females	
	Absolute[b] (95% CI)	Relative[c] %	Absolute (95% CI)	Relative %	Absolute (95% CI)	Relative %
Apr 2020	-0.4 (-0.7, 0)*	-14.1	-0.8 (-1.3, -0.3)**	-19.3	0 (-0.3, 0.3)	-1.1
May 2020	0.2 (-0.1, 0.6)	9.8	-0.1 (-0.6, 0.3)	-2.1	0.6 (0.2, 1)**	39
Jun 2020	0.4 (0.1, 0.6)**	12.8	0.5 (-0.1, 1)	12.4	0.3 (0, 0.6)	14.8
Jul 2020	0.7 (0.2, 1.2)**	24.3	0.6 (0, 1.3)	16.8	0.7 (0.2, 1.2)**	43.5
Aug 2020	0.5 (0.1, 0.9)**	18.9	0.5 (-0.1, 1.1)	13.7	0.6 (0.2, 0.9)**	32.8
Sep 2020	0.4 (-0.1, 0.8)	15.1	0.2 (-0.6, 1)	7	0.5 (0.2, 0.8)***	35.7
Oct 2020	0.2 (-0.1, 0.6)	10.3	-0.2 (-0.7, 0.3)	-2.3	0.6 (0.2, 1.1)**	42.2
Nov 2020	0.2 (-0.1, 0.5)	9.7	0.1 (-0.4, 0.6)	5	0.3 (0, 0.6)*	22.3
Dec 2020	0.1 (-0.2, 0.5)	6.7	-0.2 (-0.7, 0.4)	-2.5	0.4 (0, 0.8)*	30.3
Jan 2021	0.3 (0, 0.6)	12.2	0.2 (-0.3, 0.6)	5.4	0.4 (-0.1, 0.9)	29.8
Feb 2021	-0.1 (-0.4, 0.2)	-2.9	-0.4 (-0.9, 0.1)	-8.9	0.2 (-0.2, 0.5)	12.9
Mar 2021	0.2 (-0.2, 0.5)	6.6	-0.2 (-0.8, 0.4)	-1.6	0.5 (0.1, 0.8)*	27.8
Apr 2021	0.4 (0.1, 0.8)*	17.6	0.3 (-0.3, 0.9)	12.3	0.5 (0.2, 0.8)**	32.8
May 2021	0.4 (-0.1, 0.9)	17.6	0.2 (-0.7, 1.2)	10.4	0.6 (0.2, 1)**	37.6
Jun 2021	0.4 (0.1, 0.7)*	14.8	0.1 (-0.4, 0.7)	5.8	0.7 (0.3, 1)***	39.2
Jul 2021	0.6 (0, 1.1)*	21	0.3 (-0.5, 1.2)	10.9	0.8 (0.4, 1.2)***	48.5
Aug 2021	0.1 (-0.4, 0.7)	5	-0.3 (-1.1, 0.6)	-3.3	0.5 (0.1, 0.9)*	27.8
Sep 2021	0.4 (-0.2, 0.9)	16.2	0.2 (-0.6, 0.9)	9.2	0.5 (0.1, 1)*	36.2

*p<0.05, **p<0.01, ***p<0.001

[a] Table displays absolute and relative changes (versus predicted rates) in monthly rates of high-acuity alcohol-related liver disease complication episodes presenting to the emergency department, observation unit, or hospital.
[b] Absolute change estimates and 95% confidence intervals generated from adjusted regression model coefficients using nonlinear combinations of parameters.
[c] Relative change estimates calculated by dividing the unadjusted absolute change (unadjusted observed rate minus unadjusted predicted rate) by the unadjusted predicted rate for each pandemic month. Given that the adjusted relative changes are sensitive to the referent group chosen for each covariate in each model, we elected to calculate unadjusted relative changes.
[d] Models adjusted for member-level age category, sex, US division, seasonality, and poverty level of residence based on 5-digit zip code. Age category not included in models stratified by age and sex not included in models stratified by sex.

2. **15-39-Year-Old Subgroup**

Month[a]	Change in high-acuity alcohol-related liver disease complication episodes per 100,000 people					
	Aged 15-39		Aged 15-39, Males		Aged 15-39, Females	
	Absolute[b] (95% CI)	Relative[c] %	Absolute (95% CI)	Relative %	Absolute (95% CI)	Relative %
Apr 2020	-0.1 (-0.4, 0.1)	-12.6	-0.5 (-0.8, -0.1)*	-31	0.2 (-0.2, 0.5)	32
May 2020	0.1 (-0.2, 0.4)	16.2	0.1 (-0.4, 0.5)	10	0.2 (-0.1, 0.5)	31.6
Jun 2020	0.2 (-0.1, 0.6)	22.9	0.2 (-0.4, 0.8)	16.4	0.3 (-0.1, 0.7)	39.2
Jul 2020	0.4 (0, 0.7)*	35.4	0.4 (-0.1, 0.9)	34	0.3 (-0.1, 0.7)	39.7
Aug 2020	0.2 (-0.2, 0.5)	15.8	0.2 (-0.5, 0.8)	13.1	0.2 (-0.2, 0.5)	23.1
Sep 2020	0.1 (-0.3, 0.5)	11.1	0 (-0.7, 0.6)	-0.5	0.2 (-0.2, 0.5)	39.7
Oct 2020	0 (-0.3, 0.4)	8.5	0 (-0.5, 0.6)	2.7	0.1 (-0.5, 0.6)	23.1
Nov 2020	0 (-0.4, 0.4)	0.7	0 (-0.6, 0.6)	2.1	-0.1 (-0.5, 0.3)	-2
Dec 2020	0 (-0.2, 0.3)	2.6	-0.2 (-0.7, 0.3)	-11.9	0.3 (-0.2, 0.7)	38.2
Jan 2021	0.2 (-0.1, 0.6)	20.6	-0.3 (-1, 0.4)	-18	0.8 (0.2, 1.3)**	114.1
Feb 2021	-0.1 (-0.4, 0.3)	-9	-0.6 (-1.1, -0.1)*	-38.9	0.4 (-0.1, 1)	63.7
Mar 2021	-0.1 (-0.5, 0.2)	-9.8	-0.2 (-0.7, 0.4)	-5.9	-0.1 (-0.5, 0.2)	-18.9
Apr 2021	0.3 (0, 0.6)	30.8	0.1 (-0.4, 0.7)	17.3	0.4 (-0.1, 0.9)	64.5
May 2021	0.2 (-0.2, 0.5)	22.8	0.1 (-0.4, 0.6)	15.5	0.3 (-0.2, 0.7)	41.8
Jun 2021	0.3 (-0.2, 0.8)	29.9	0.1 (-0.5, 0.6)	10.4	0.5 (-0.1, 1.2)	78.2
Jul 2021	0.1 (-0.3, 0.5)	10.1	0 (-0.7, 0.6)	2.1	0.2 (-0.2, 0.7)	30.6

© 2024 Shuey B et al. *JAMA Health Forum.*

Aug 2021	-0.1 (-0.5, 0.3)	-5.9	-0.5 (-1, 0.1)	-25.5	0.3 (-0.1, 0.7)	42.4
Sep 2021	0.1 (-0.2, 0.4)	15.3	0.3 (-0.4, 0.9)	22	-0.1 (-0.6, 0.4)	0.6

*p<0.05, **p<0.01, ***p<0.001

[a] Table displays absolute and relative changes (versus predicted rates) in monthly rates of high-acuity alcohol-related liver disease complication episodes presenting to the emergency department, observation unit, or hospital.
[b] Absolute change estimates and 95% confidence intervals generated from adjusted regression model coefficients using nonlinear combinations of parameters.
[c] Relative change estimates calculated by dividing the unadjusted absolute change (unadjusted observed rate minus unadjusted predicted rate) by the unadjusted predicted rate for each pandemic month. Given that the adjusted relative changes are sensitive to the referent group chosen for each covariate in each model, we elected to calculate unadjusted relative changes.
[d] Models adjusted for member-level age category, sex, US division, seasonality, and poverty level of residence based on 5-digit zip code. Age category not included in models stratified by age and sex not included in models stratified by sex.

3. 40–64-Year-Old Subgroup

Change in high-acuity alcohol-related liver disease complication episodes per 100,000 people

Month[a]	Aged 40-64		Aged 40-64, Males		Aged 40-64, Females	
	Absolute[b] (95% CI)	Relative[c] %	Absolute (95% CI)	Relative %	Absolute (95% CI)	Relative %
Apr 2020	-0.3 (-1, 0.4)	-9.1	-0.9 (-1.9, 0.2)	-15.1	0.3 (-0.4, 1)	6.4
May 2020	1 (0.4, 1.7)**	24.6	0.3 (-0.6, 1.3)	7	1.7 (0.9, 2.6)***	69.1
Jun 2020	0.9 (0.1, 1.7)*	21	1 (-0.4, 2.3)	16.2	0.8 (0.1, 1.5)*	34.1
Jul 2020	1.5 (0.6, 2.5)**	37	1.7 (0.3, 3)*	29	1.3 (0.3, 2.4)*	58.4
Aug 2020	1.4 (0.4, 2.5)**	35.1	1.6 (0.1, 3)*	26.8	1.3 (0.5, 2.2)**	57.5
Sep 2020	0.9 (0, 1.8)*	21.5	0.4 (-1.3, 2.1)	7.3	1.5 (1, 2.1)***	58.9
Oct 2020	1.1 (0.3, 1.8)**	24.9	0.3 (-0.8, 1.5)	6.9	1.8 (0.8, 2.8)***	72.2
Nov 2020	0.6 (-0.1, 1.3)	13.9	0.1 (-1.3, 1.5)	2.9	1.2 (0.4, 2)**	43.5
Dec 2020	0.3 (-0.4, 1)	9.8	-0.3 (-1.4, 0.7)	-3.9	0.9 (0.1, 1.6)*	46.5
Jan 2021	0.9 (0.2, 1.6)*	26.1	1.1 (0.1, 2)*	20.6	0.8 (-0.1, 1.6)	41.9
Feb 2021	0.6 (0, 1.3)	18.7	0.3 (-0.7, 1.3)	6.4	1.0 (0.1, 1.9)*	52.3
Mar 2021	1.4 (0.5, 2.3)**	33.7	1.1 (-0.1, 2.3)	21.1	1.6 (0.7, 2.6)***	68.5
Apr 2021	0.9 (0.1, 1.8)*	24.1	0.6 (-1, 2.1)	14.2	1.3 (0.5, 2)**	52.6
May 2021	1.7 (0.6, 2.8)**	42.7	1.8 (-0.2, 3.7)	34.4	1.6 (0.7, 2.5)***	67.7
Jun 2021	1.3 (0.7, 2)**	34.5	1 (-0.1, 2.2)	20.3	1.6 (1, 2.3)***	75.3
Jul 2021	1.7 (0.4, 3)**	43.6	1.4 (-0.7, 3.4)	25.8	2.1 (0.9, 3.2)***	94.7
Aug 2021	0.7 (-0.6, 2.1)	19.2	0.2 (-1.8, 2.3)	6.2	1.2 (0.1, 2.4)*	56.9
Sep 2021	1.3 (0, 2.6)	31.9	0.6 (-1.1, 2.2)	13.1	2.0 (0.8, 3.2)***	85.8

*p<0.05, **p<0.01, ***p<0.001

[a] Table displays absolute and relative changes (versus predicted rates) in monthly rates of high-acuity alcohol-related liver disease complication episodes presenting to the emergency department, observation unit, or hospital.
[b] Absolute change estimates and 95% confidence intervals generated from adjusted regression model coefficients using nonlinear combinations of parameters.
[c] Relative change estimates calculated by dividing the unadjusted absolute change (unadjusted observed rate minus unadjusted predicted rate) by the unadjusted predicted rate for each pandemic month. Given that the adjusted relative changes are sensitive to the referent group chosen for each covariate in each model, we elected to calculate unadjusted relative changes.
[d] Models adjusted for member-level age category, sex, US division, seasonality, and poverty level of residence based on 5-digit zip code. Age category not included in models stratified by age and sex not included in models stratified by sex.

4. 65-74-Year-Old Subgroup

Change in high-acuity alcohol-related liver disease complication episodes per 100,000 people

Month[a]	Aged 65-74		Aged 65-74, Males		Aged 65-74, Females	
	Absolute[b] (95% CI)	Relative[c] %	Absolute (95% CI)	Relative %	Absolute (95% CI)	Relative %
Apr 2020	-0.8 (-1.6, 0)*	-19	-1.4 (-2.7, -0.1)*	-20.7	-0.3 (-1, 0.5)	-15.6
May 2020	-0.6 (-1.4, 0.2)	-14.3	-1.1 (-2.4, 0.1)	-15.7	-0.2 (-1.1, 0.7)	-11.4
Jun 2020	0.3 (-0.4, 1)	2.4	0.7 (-0.7, 2)	9.9	0 (-0.7, 0.6)	-15.3
Jul 2020	0.5 (-0.3, 1.4)	8.8	0 (-1.1, 1.1)	-0.7	1 (0.1, 1.8)*	30.3

© 2024 Shuey B et al. *JAMA Health Forum.*

Aug 2020	0.3 (-0.6, 1.2)	2.1	0.2 (-1.4, 1.7)	1.7	0.4 (-0.4, 1.1)	3
Sep 2020	0.2 (-0.8, 1.3)	8.8	0.5 (-1.4, 2.3)	8.4	0 (-0.8, 0.8)	9.4
Oct 2020	-0.6 (-1.3, 0.1)	-10.9	-1.2 (-2.7, 0.3)	-18.1	-0.1 (-0.7, 0.6)	5.5
Nov 2020	-0.6 (-1.3, 0)	-11.9	-0.6 (-1.9, 0.7)	-8.1	-0.6 (-1.3, 0)	-20.6
Dec 2020	-0.1 (-1.1, 0.8)	-3.9	-0.1 (-1.8, 1.5)	-2.5	-0.1 (-0.8, 0.6)	-6.9
Jan 2021	-0.5 (-1.3, 0.3)	-11.5	-0.7 (-1.9, 0.5)	-10.6	-0.3 (-1.1, 0.5)	-14.9
Feb 2021	-1 (-1.7, -0.3)**	-24.3	-1.5 (-2.7, -0.2)	-22.1	-0.7 (-1.3, 0)*	-30.3
Mar 2021	-0.9 (-1.8, 0)*	-19.8	-1.8 (-3.1, -0.5)**	-24.2	-0.2 (-1, 0.6)	-10.8
Apr 2021	0.1 (-0.8, 1)	3.4	-0.1 (-1.5, 1.3)	1.6	0.2 (-0.6, 1)	6.1
May 2021	-0.6 (-1.6, 0.5)	-11.2	-1.3 (-3.1, 0.5)	-16.2	0 (-1, 1.1)	-0.9
Jun 2021	-0.2 (-1.1, 0.7)	-9.2	-0.5 (-2, 0.9)	-8.3	0 (-0.8, 0.9)	-12.3
Jul 2021	0.3 (-0.8, 1.3)	1.9	-0.3 (-1.9, 1.4)	-4.2	0.7 (-0.3, 1.6)	14.6
Aug 2021	-0.4 (-0.9, 0.2)	-12.5	-0.8 (-2, 0.4)	-12	0 (-0.7, 0.6)	-14.7
Sep 2021	-0.1 (-0.9, 0.8)	2	0 (-1.7, 1.6)	1.1	-0.1 (-0.8, 0.7)	3.3

*p<0.05, **p<0.01, ***p<0.001

[a] Table displays absolute and relative changes (versus predicted rates) in monthly rates of high-acuity alcohol-related liver disease complication episodes presenting to the emergency department, observation unit, or hospital.
[b] Absolute change estimates and 95% confidence intervals generated from adjusted regression model coefficients using nonlinear combinations of parameters.
[c] Relative change estimates calculated by dividing the unadjusted absolute change (unadjusted observed rate minus unadjusted predicted rate) by the unadjusted predicted rate for each pandemic month. Given that the adjusted relative changes are sensitive to the referent group chosen for each covariate in each model, we elected to calculate unadjusted relative changes.
[d] Models adjusted for member-level age category, sex, US division, seasonality, and poverty level of residence based on 5-digit zip code. Age category not included in models stratified by age and sex not included in models stratified by sex.

5. 75-Year-Old and Older Subgroup

Month[a]	Change in high-acuity alcohol-related liver disease complication episodes per 100,000 people					
	Aged 75+		Aged 75+, Males		Aged 75+, Females	
	Absolute[b] (95% CI)	Relative[c] %	Absolute (95% CI)	Relative %	Absolute (95% CI)	Relative %
Apr 2020	-0.4 (-0.9, 0.1)	-30.3	-0.6 (-1.6, 0.4)	-28.9	-0.3 (-0.7, 0.1)	-34.1
May 2020	0 (-0.5, 0.5)	-1	-0.5 (-1.3, 0.3)	-25.1	0.3 (-0.2, 0.9)	59.2
Jun 2020	-0.2 (-0.6, 0.2)	-14.5	-0.4 (-1.3, 0.6)	-13.9	-0.1 (-0.5, 0.2)	-16.2
Jul 2020	-0.2 (-0.6, 0.2)	-12.2	-0.5 (-1.2, 0.3)	-17.8	0 (-0.4, 0.5)	1.7
Aug 2020	-0.2 (-0.7, 0.3)	-15	-0.7 (-1.6, 0.3)	-25.2	0.1 (-0.4, 0.5)	10.4
Sep 2020	-0.1 (-0.6, 0.3)	-7.1	-0.3 (-1.4, 0.7)	-6.5	0 (-0.3, 0.3)	-8.5
Oct 2020	-0.1 (-0.5, 0.4)	-1.9	-0.5 (-1.5, 0.4)	-13.9	0.3 (-0.3, 0.8)	27.6
Nov 2020	0.8 (0.1, 1.5)*	56.3	1 (-0.5, 2.4)	46.2	0.7 (0.1, 1.2)*	81.4
Dec 2020	0.2 (-0.2, 0.7)	13.9	0.1 (-0.7, 0.9)	-2.4	0.4 (-0.2, 0.9)	53.5
Jan 2021	0.2 (-0.2, 0.5)	9.4	0.1 (-0.9, 1.1)	-0.7	0.2 (-0.4, 0.8)	32.7
Feb 2021	-0.4 (-0.9, 0)	-29.6	-0.3 (-1.2, 0.6)	-16.9	-0.5 (-0.8, -0.2)**	-61.3
Mar 2021	-0.3 (-0.8, 0.2)	-18.6	-0.8 (-1.9, 0.3)	-36.1	0.1 (-0.3, 0.5)	23
Apr 2021	0.1 (-0.5, 0.7)	6.1	0.4 (-1, 1.8)	11.7	-0.1 (-0.6, 0.3)	-8.3
May 2021	-0.2 (-0.8, 0.3)	-14.2	-0.7 (-1.7, 0.4)	-29.6	0.1 (-0.5, 0.7)	22
Jun 2021	-0.3 (-0.7, 0)	-20.9	-1 (-1.7, -0.2)*	-35.9	0.1 (-0.3, 0.5)	14.1
Jul 2021	-0.3 (-0.8, 0.1)	-20.8	-0.5 (-1.5, 0.4)	-19.6	-0.2 (-0.6, 0.3)	-24
Aug 2021	-0.1 (-0.7, 0.4)	-7	-0.5 (-1.7, 0.6)	-19.4	0.2 (-0.4, 0.7)	21.4
Sep 2021	-0.4 (-1, 0.1)	-25	-0.8 (-1.8, 0.2)	-22.3	-0.2 (-0.7, 0.3)	-31.8

*p<0.05, **p<0.01, ***p<0.001

[a] Table displays absolute and relative changes (versus predicted rates) in monthly rates of high-acuity alcohol-related liver disease complication episodes presenting to the emergency department, observation unit, or hospital.
[b] Absolute change estimates and 95% confidence intervals generated from adjusted regression model coefficients using nonlinear combinations of parameters.
[c] Relative change estimates calculated by dividing the unadjusted absolute change (unadjusted observed rate minus unadjusted predicted rate) by the unadjusted predicted rate for each pandemic month. Given that the adjusted relative changes are sensitive to the referent group chosen for each covariate in each model, we elected to calculate unadjusted relative changes.
[d] Models adjusted for member-level age category, sex, US division, seasonality, and poverty level of residence based on 5-digit zip code. Age category not included in models stratified by age and sex not included in models stratified by sex.
