# Supplementary material for: High-Acuity Alcohol-Related Complications During the COVID-19 Pandemic
Source: JAMA Health Forum. 2024 Apr 12;5(4):e240501. doi: 10.1001/jamahealthforum.2024.0501 (PMC11065164; doi:10.1001/jamahealthforum.2024.0501)
Supplement: Supplement 2. — Data Sharing Statement [file jamahealthforum-e240501-s002.pdf]

Data Sharing Statement

Shuey. High-Acuity Alcohol-Related Complications During the COVID-19 Pandemic. *JAMA Health Forum*. Published April 12, 2024. doi:10.1001/jamahealthforum.2024.0501

Data
Data available: No

Additional Information
Explanation for why data not available: Person-level data cannot be made available to others due to data use agreements. By request to the authors, statistical and analytic code can be made available.
